# Peripheral Inflammation and Sleep Loss Induce Coordinated Motivational Changes: An Experimental Two‐Hit Stress Model

**DOI:** 10.1111/jsr.70335

**Published:** 2026-04-27

**Authors:** John Axelsson, Mats Lekander, Camilla I. Svensson, Nils Simon, Alexandra Kuliszkiewicz, Julie Lasselin, Leonie J. T. Balter

**Affiliations:** ^1^ Department of Psychology Stress Research Institute, Stockholm University Stockholm Sweden; ^2^ Department of Clinical Neuroscience, Division of Psychology Karolinska Institutet Stockholm Sweden; ^3^ Osher Center for Integrative Health, Karolinska Institutet Stockholm Sweden; ^4^ Department of Physiology and Pharmacology Center for Molecular Medicine, Karolinska Institutet Stockholm Sweden; ^5^ Department of Psychiatry Radboud University Nijmegen Medical Centre Nijmegen the Netherlands; ^6^ Donders Institute for Brain, Cognition and Behaviour, Radboud University Nijmegen Nijmegen the Netherlands

**Keywords:** daily life behaviours, experimental, low‐grade inflammation, motivation, sleep loss

## Abstract

Sleep loss and low‐grade inflammation are common stressors that affect behaviour and recovery needs. To examine their separate and combined effects on motivation for everyday behaviours, we developed a two‐hit experimental model. Participants (*N* = 56, 31 women) completed three experimental visits: sleep saturation (two nights, 9 h in bed/night); sleep‐restriction (two nights, 4 h in bed/night); and an immune challenge (~24 h post COVID‐19 vaccination) combined with either sleep saturation (*n* = 26) or restriction (*n* = 30). Motivation for everyday behaviours and sleepiness were rated, and blood samples were collected. Both sleep restriction and vaccination increased motivation for sleep‐related behaviours and reduced motivation for physical and social activity, with sleep restriction having broader effects. These changes were associated with inflammation and sleepiness. Vaccination increased thirst, while sleep restriction reduced it, but neither affected hunger or food preferences. Sleep restriction amplified the inflammatory response to vaccination. For motivation, the stressors combined caused larger changes than either alone for some outcomes, without amplifying one another. These findings support that different stressors reallocate motivation towards rest and sleep‐supporting behaviours over physical and social activity, likely to drive behaviours that support homeostasis after allostatic challenges. As these stressors often co‐occur, this adaptive response can meaningfully shape daily functioning. Future work may determine when this functional response becomes maladaptive.

## 1 Introduction

Across species, behavioural reorganisation during periods of sickness is a well‐conserved adaptive response (Hart 1988). Sickness is typically characterised by heightened inflammation levels (Dantzer et al. 2008), which are associated with a set of motivational and behavioural changes, including fatigue, anhedonia and reduced appetite. These changes are believed to function as motivational signals that reorganise behaviour towards energy conservation and recovery during sickness and inflammatory states (Hart 1988; Dantzer et al. 2008; Dantzer and Kelley 2007). However, when sustained or repeatedly triggered, these shifts in motivation, and the resulting behaviours, may also increase vulnerability to social isolation and physical inactivity, contributing to cardiovascular diseases and mental health problems over longer timeframes (Lee et al. 2012; Matthews and Tye 2019).

Sleep loss and sleepiness also profoundly impact behavioural motivation. Individuals deprived of a full night of sleep exhibited strong motivation for rest and safety‐seeking behaviours, while showing reduced motivation for social and physical activities (Axelsson et al. 2020), and more generally promoting social withdrawal and avoidance (Ben Simon and Walker 2018). In a naturalistic setting, subjective sleepiness predicted reduced social activity during the subsequent 3 h (Holding et al. 2020). At the same time, there is evidence that individuals may temporarily increase engagement in stimulating physical or social activities as a countermeasure to fatigue or sleepiness. Such strategies have been documented primarily in short‐term, high‐demand, or task‐constrained contexts where disengagement is no option (e.g., pilots during active flight operations) (Caldwell et al. 2009). Together, these findings support a model where sleepiness and inflammation act as motivational signals that coordinate behaviour to prioritise rest and sleep. Despite these parallels, the extent to which inflammation‐ and sleep loss‐induced motivational changes overlap, interact, or diverge remains poorly understood. This is of particular relevance as physiological stressors rarely occur in isolation. Indeed, stressors are abundant, often co‐exist in everyday life, and may amplify each other's effect (McEwen and Karatsoreos 2015). Sleep disturbances are common in various disorders characterised by low‐grade inflammation (Ditmer et al. 2022; Irwin et al. 2016). At the same time, inflammatory states modulate sleep (Besedovsky et al. 2019), often including longer sleep (Lasselin et al. 2019), reduced rapid eye movement (REM) sleep (Opp and Krueger 2015), and more frequent awakenings (Sharpley et al. 2016). Thus, studying sleep and inflammation in conjunction could yield valuable insights into how these physiological stressors interact, knowledge that, in turn, can inform research into the development of targeted interventions.

Both inflammation and prolonged wakefulness are allostatic challenges that are energetically demanding (Lacourt et al. 2018; Lesku and Schmidt 2022; Wang and Medzhitov 2019) and it is plausible that they influence motivation in similar ways—promoting behaviours that encourage recovery. A desire to rest and sleep and avoidance of physical and social activities would support recovery from both these physiological challenges. However, their effects on other domains may diverge, such as food and thirst. Inflammation and sickness typically reduce the motivation to eat (Hart 2009), while sleep loss may enhance or minimally affect the motivation to eat (Axelsson et al. 2020; Greer et al. 2013; Kracht et al. 2019). Regarding social motivation, acute inflammation enhanced affiliative motivation towards close others and amplified response in the ventral striatum when presented with images of close others compared to strangers (Inagaki et al. 2015). Sleep loss has been found to impact social relationships in a partially similar way—reducing willingness to interact with other people, including both strangers and friends, while increasing willingness to receive care from familiar people (Axelsson et al. 2020). Other studies also found that sleep loss leads to social withdrawal (Ben Simon and Walker 2018; Gordon et al. 2021), however these studies did not specifically examine the impact on closer social relationships. It is unclear how inflammation and sleep loss overlap and differ in their motivational impact. Social withdrawal and inactivity are implicated in a range of disorders, from depression to cardiovascular diseases and chronic fatigue syndrome (Cacioppo et al. 2006; Pearce et al. 2022; Valtorta et al. 2016), and they impede effective treatments and clinical outcomes (Khazanov et al. 2020; Wang et al. 2020). Understanding how common physiological stressors like inflammation and insufficient sleep influence motivation is therefore valuable for identifying factors that shape engagement in social and physical activities in everyday life.

In the present study, we used an experimental cross‐over design to determine how sleep restriction and an inflammatory immune challenge, alone and in combination, impact self‐rated motivation for everyday behaviours. Subjective motivation remains largely understudied in the sleep and inflammation field, despite its significance for driving observable behavioural changes. Using a COVID‐19 vaccine as a novel inflammatory stimulus, we assessed self‐rated motivation, sleepiness and blood‐based inflammatory markers. We sought to determine whether these factors amplify each other's effect and their associations with peripheral levels of inflammatory cytokines and sleepiness levels. We hypothesised that both the inflammatory stimulus and sleep loss would increase the desire for safety‐seeking behaviours, sleep prioritisation, and willingness to allocate resources to be allowed to rest. Concurrently, we expected a decrease in motivation for other competing activities such as physical activities and social activities with unfamiliar people, but less so for familiar people (friends). Motivation for eating a meal and hunger were expected to decrease following the inflammatory stimulus. With respect to sleep loss, meta‐analyses show that sleep restriction increases daily caloric intake, hunger and weight gain (Zhu et al. 2019). Most studies assess eating behaviour under ad libitum food availability, and the effects appear less robust in real world contexts (Dickinson et al. 2025). In a prior study examining the effects of sleep deprivation on motivation for different food categories and subjective hunger, hunger was only modestly increased, while motivation for specific foods (fruit, sweets/candy, or steak/protein) remained unaffected (Axelsson et al. 2020). These findings suggest that sleep loss may influence eating behaviour in certain contexts, but without robustly increasing self‐reported food motivation. To test these hypotheses, participants completed three conditions: sleep saturation (2 nights with 9 h/bed); sleep restriction (2 nights with 4 h/bed); and a COVID‐19 vaccine condition (inducing a low‐grade inflammatory state) combined with either sleep restriction or sleep saturation.

## 2 Materials and Methods

### 2.1 Participants

A total of 56 participants (31 women, 25 men, 0 non‐binary/other; *M*age = 25.5 years, SD = 4.9 years, range 18–39 years) were invited to complete three experimental conditions in a pseudorandomized order: (1) sleep saturation at home (two nights with instructed 9 h in bed/night, *n* = 53), (2) sleep restriction at home (two nights with instructed 4 h in bed/night, *n* = 50), and (3) a COVID‐19 vaccination in combination with either sleep saturation (*n* = 26; 14 women, 12 men) or sleep restriction (*n* = 30; 17 women, 13 men). See Figure 1 for the design and Supplement for details on the rationale for the chosen experimental manipulations. Participants were recruited via university recruitment systems, social media adverts and public flyers. The mean BMI was 23.4 (SD = 4.6, range 14.3–36.5, *M* = 23.3 for women and *M* = 23.4 for men) and body fat percentage was 30.4% (SD = 8.8, range 8.0–48.6) for women and 18.6% (SD = 5.6, range 6.9–29.5) for men. Fifty participants completed all three conditions, three participants completed the vaccine condition only, and three participants completed the vaccine condition and one sleep condition (two with sleep saturation, one with sleep restriction), resulting in a total of 159 completed visits. Exclusion criteria were not being fluent in Swedish or English, habitual sleep outside the 7–9 h range, current sleep disorder or serious psychiatric disorder, having worked night shifts or travelled three or more time zones in the 3 weeks prior to each visit, problems with abstaining from caffeine‐containing products for 1 day, and intake of medication affecting sleep or sleepiness. Three participants came for their first vaccine dose, 38 for their second, and 15 for their third. See Table S1 for information on vaccination history and conditions. For analyses involving sleep condition, data were excluded when participants did not adhere properly to the sleep protocol. After exclusion, there were 45 sleep saturation no‐vaccine sessions, 26 sleep restriction no‐vaccine sessions, 21 sleep saturation vaccine sessions, and 21 sleep restriction vaccine sessions. Details can be found in the “Data exclusion/inclusion” section below. This study was approved by the Swedish Ethical Review Authority (dnr: 2021‐03730). Participants provided written informed consent and received monetary compensation.

**FIGURE 1:**
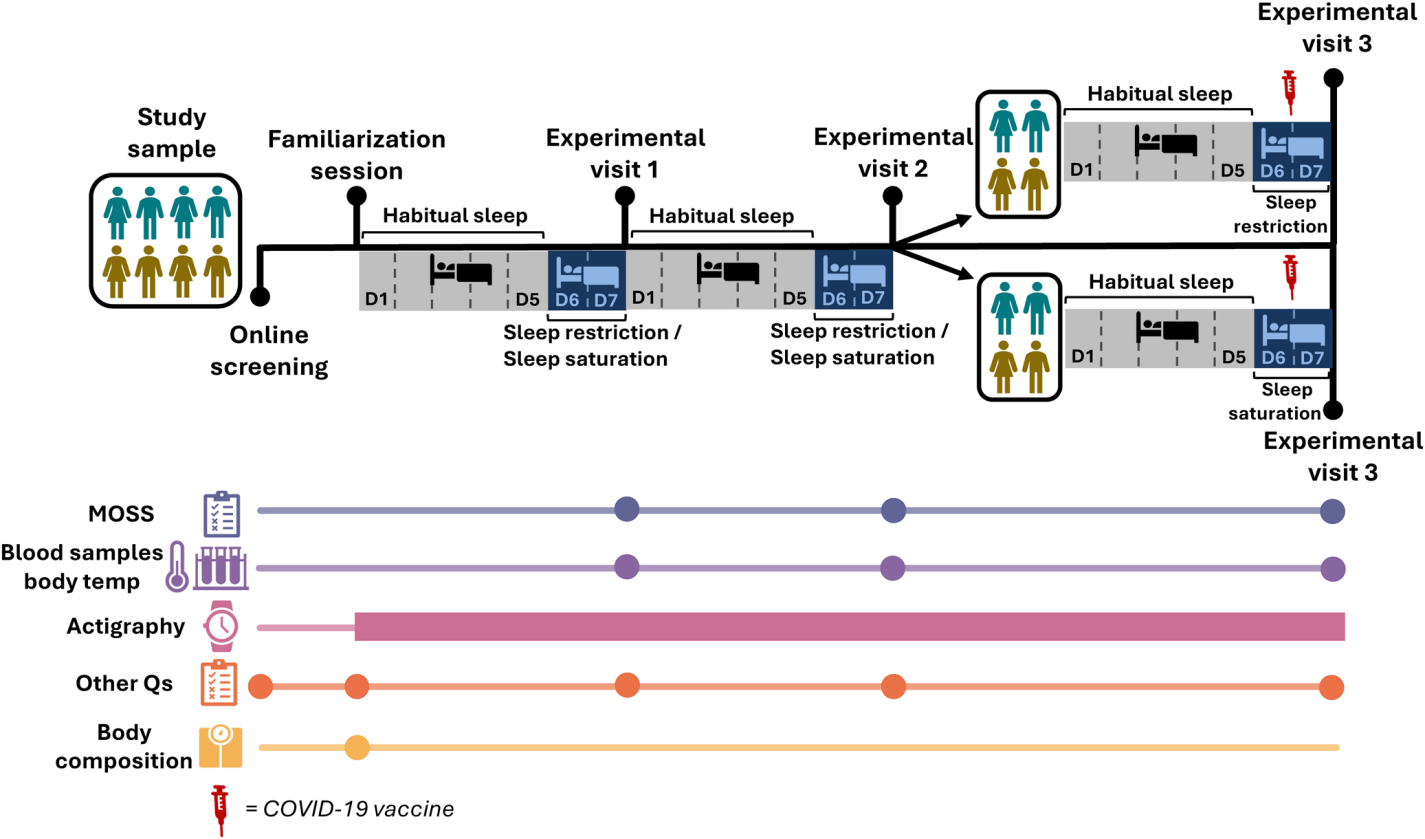
Study design and timeline. All participants completed four in‐lab sessions: One familiarisation session followed by three experimental visits. Experimental visits included two non‐vaccine sessions under two sleep conditions (Sleep restriction: 2 nights with instructed 4 h in bed/night and Sleep saturation: 2 nights with instructed 9 h in bed/night), and one vaccine session combined with either Sleep restriction or Sleep saturation (pseudorandomized between participants). D = Day, MOSS = Motivation Scale of Sleepiness; Other Qs = Other questionnaires.

### 2.2 Procedures

Initial eligibility was assessed through an online screening questionnaire. Eligible participants were then invited to four in‐lab sessions: one familiarisation session and three experimental visits. During the familiarisation session, participants completed baseline questionnaires, practised tests, and were informed of their assigned condition order. The three experimental visits included two non‐vaccine sessions conducted under both sleep conditions (sleep restriction, sleep saturation), in a within‐subjects design. A third session involved administration of a COVID‐19 vaccine and was combined with either sleep restriction or sleep saturation. The order of the three sessions was counterbalanced across participants. Starting 7 days prior the experimental visits, participants kept an electronic sleep diary (Karolinska Sleep Diary (Akerstedt et al. 1994), completed via a smartphone application) and wore a wrist‐worn actigraph (ActTrust 2, Condor Instruments, Brazil) to measure sleep and physical activity throughout the protocol. The full protocol lasted a minimum of 3 weeks, with at least a seven‐day period between each experimental visit to allow for sufficient recovery from both sleep restriction and immune activation from vaccination. Participants slept normally for 5 days followed by two nights according to the agreed sleep protocol. While participants were instructed to be in bed for 9 h or 4 h/night for two nights, the sleep protocol timings (for the sleep saturation and sleep restriction conditions) were tailored to the individual's habitual sleep timings. Bed and wake times were required to remain within a three‐hour window of their usual timings and within 3 h between conditions. After two nights following the assigned sleep protocol, participants came for a lab visit. The lab visits were scheduled either in the morning (starting 8:30–9:30) or in the afternoon (starting 12:30–13:30). Each participant completed the lab visits either in the morning or in the afternoon. To avoid a strong alignment between time of testing and self‐reported chronotype (i.e., morning types primarily being tested in the morning and evening chronotypes primarily in the afternoon), session assignment was guided by the participant's chronotype (assessed using a single‐item question during the screening) and their availability (e.g., work, study or travel). Of the 56 participants, 21 identified as morning types, 15 as neither, and 20 as evening types. Thirty were assigned to the morning session (8 morning chronotypes, 10 neither, 12 evening chronotypes), and 26 to the afternoon (13 morning chronotypes, 5 neither, 8 evening chronotypes). Participants were instructed to avoid naps, alcohol, strenuous exercise and caffeinated drinks for 24 h prior to the experimental visit. Participants arrived fasted (morning session) or after abstaining from food for at least 4 h (afternoon session). Before breakfast or lunch was provided, motivation for everyday life behaviours was assessed using the motivation scale of sleepiness (MOSS), body temperature was measured, and a blood sample was taken. These assessments were scheduled to occur ~24 h post‐vaccination (range 19 h30–28 h30 post‐vaccination). Participants selected their own vaccination provider and provided their proof of vaccination during the lab visit. Vaccinations were administered as part of standard clinical practise in accordance with public health guidelines, without delaying or altering participants' access to recommended COVID‐19 vaccination schedules. The vaccine was administered during the daytime between the two consecutive days of their assigned sleep condition (sleep restriction night‐vaccination‐sleep restriction night or sleep saturation night‐vaccination‐sleep saturation night). The sleep condition within the vaccine condition was pseudorandomized to ensure a similar distribution of participants who received their first, second, or third vaccine dose. For safety reasons, as sleep restriction was expected to induce sleepiness, participants were instructed to use public transportation or alternative travel arrangements when travelling to the testing facilities.

#### 2.2.1 Sleep

Time in bed and total sleep time were assessed using ActTrust 2 actigraphs, which recorded activity (proportional integration mode (PIM) levels based on 3‐axis accelerometer), light, temperature and event markers (participants pressed a button at lights out and when rising from bed) in 30‐s epochs. Data were processed in ActStudio software (Condor Instruments, Brazil). A trained sleep technician manually scored sleep and wake epochs based on patterns in activity, light levels, temperature fluctuations and event markers. Sleep timing was further reviewed and verified against participants' sleep diaries to improve scoring accuracy. Post hoc, actigraphy data were used to evaluate adherence to the sleep protocol, as it provides an objective assessment of sleep timing. We observed discrepancies between the electronic sleep diary and actigraphy with respect to reported bedtimes and wake times, which resulted in inconsistent estimates of time in bed. Therefore, actigraphy was used as the primary measure for determining protocol compliance during the experimental nights.

#### 2.2.2 Composite Inflammation Score

Fasting plasma samples were collected using ethylenediaminetetraacetic acid (EDTA) vacutainers. Samples were centrifuged at 1500 g for 10 min at 4°C and plasma was aliquoted and stored at −80°C for later assessment. Eighteen cytokines and chemokines were analysed by immunoassay (Meso Scale Discovery, Rockville, MD, USA). We constructed a composite inflammation index from all measured markers included in the custom‐built V‐PLEX Custom Proinflammatory Panel 1 (Meso Scale Discovery, Rockville, MD, USA), which included interferon gamma (IFN‐γ), interleukin (IL)‐6, IL‐8, IL‐10, and tumour necrosis factor alpha (TNF‐α). We also included CRP, which was quantified as part of the V‐PLEX Custom Human Vascular Injury panel (Meso Scale Discovery, Rockville, MD, USA). These markers are key mediators of the systemic inflammatory response and frequently studied in psychoneuroimmunology research (e.g., Dowlati et al. 2010; Felger et al. 2020). In addition to these, 12 further cytokines and chemokines were assessed based on prior evidence linking some of them to an effective immune response to COVID‐19 vaccines (Bergamaschi et al. 2021). These additional markers were: IL‐7, IL‐12/23p40, IL‐15, IL‐16, and vascular endothelial growth factor (VEGF) from the Cytokine panel 1; Eotaxin, macrophage inflammatory protein 1 alpha (MIP‐1a), MIP‐1beta (MIP‐1β), interferon gamma‐induced protein 10 (IP‐10), monocyte chemoattractant protein 1 (MCP‐1), macrophage‐derived chemokine (MDC) from the Chemokine panel 1; and serum amyloid A (Human Vascular Injury). Results for these markers are not included in the composite index. Each log transformed biomarker was *z*‐standardised prior to averaging to create a composite inflammation score, referred to as inflammation score for the remainder of the text. Analyses were performed according to manufacturer instructions. Detailed assay procedures can be found in the product insert, available at: https://www.mesoscale.com/products/v‐plex‐proinflammatory‐panel‐1‐human‐kit‐k15049d/ under *Documentation* > *Product Insert*.

#### 2.2.3 Body Temperature

Tympanic body temperature was measured in degrees Celsius using an ear thermometer (ThermoScan).

#### 2.2.4 Sleepiness

Subjective sleepiness was assessed using the Karolinska Sleepiness Scale (KSS). This is a well‐validated scale where responses are strongly affected by sleep loss and relate to physiological EEG changes (Åkerstedt and Gillberg 1990).

#### 2.2.5 Motivation

The 23‐item motivation scale of sleepiness (MOSS) was completed at the beginning of the lab visit, prior to collecting the fasting blood sample. MOSS assesses the motivation for different behaviours (Axelsson et al. 2020), covering dimensions of sleep preparation, sleep, social activities, physical activities and recreation, resource utilisation, and food preferences. We made slight modifications to the scale. Three items were added and three items were removed. See Supplement for the full questionnaire and changes. The item “How much do you want to be taken care of by your partner right now?” was only analysed for participants who indicated having a partner (*n* = 32). The scale can be found in Table S2. Internal consistency was high (*ω*_total_ = 0.90). A bifactor model indicated that there is meaningful multidimensionality with a large proportion of reliable variance being specific to the subscales (*ω*_hierarchical_ = 0.53; Explained Common Variance (ECV) = 0.26).

#### 2.2.6 Anthropometric Measures

Participants were asked to remove footwear and coats and empty their pockets before height and a body composition measurement was taken using an impedance measure (TANITA BC‐418, Tanita Europe, Amsterdam, the Netherlands). Body mass index (BMI) was included for the current analyses.

### 2.3 Statistical Approach

To examine the impact of vaccine and sleep condition on subjective motivation, inflammation score, sleepiness level, body temperature and actigraphy outcomes (time in bed and total sleep time) mixed‐effects models were fitted using the *lmer* function of the lme4 R package (Bates et al. 2015). Given the clustered structure of the data, all models included a random intercept for participant ID. To identify the best‐fitting model for each outcome, we performed stepwise model selection using the *Buildmer* R package (Voeten 2020) (except for the actigraphy analyses). The procedure began with a full model including all fixed effects of interest (vaccine condition, sleep condition and their interaction, specified as dv ~ vaccine condition (coded 0 = no vaccine, 1 = vaccine) * sleep condition (coded 0 = sleep saturation, 1 = sleep restriction) + (1 | ID) and retained the random intercept for participant ID throughout. Using backward selection, fixed effects were removed iteratively when their exclusion did not significantly worsen model fit, as determined by a likelihood ratio test. To assess correlations between motivation items and physiological measures (inflammation scores and sleepiness) across all conditions, mixed‐effects models were fitted with each physiological measure as a fixed effect predictor, including a random intercept for participant ID (item ~ sleepiness + (1 | ID)). Adjusted models additionally added gender (coded 0 = woman, 1 = man), body mass index (BMI), vaccine dose (continuous 1, 2, or 3), and time of testing (coded 0 = morning, 1 = afternoon), specified as item ~ sleepiness + gender + BMI + vaccine dose + time of testing + (1 | ID)). The results of the adjusted models are reported in the Supporting Information. Two items of the MOSS relating to utilisation of energy resources (walk and paying money in return for being allowed to go to bed) were rank transformed to address skewness and outliers. All other MOSS items were rated on a 5‐point scale.

The *omega* function of the psych R package was used to estimate reliability indices for the MOSS (Revelle 2025). *ω*_total_ quantifies the proportion of total‐score variance that is reliable (1‐*ω*_total_ reflects random error). The *ω*_hierarchical_ is the proportion of total‐score variance attributable to a single general factor in a bifactor model. Values of ≥ 0.70 are considered desired if the scale is treated as unidimensional. The ECV is a measure to judge the degree of unidimensionality by indexing the share of the common variance accounted for by the general factor. ECV values ≥ 0.60 are often suggested as supporting a unidimensional score (Rodriguez et al. 2016).

#### 2.3.1 Data Inclusion/Exclusion

All participants who completed the vaccine condition (*n* = 56) were included in the analyses examining the associations between MOSS items, inflammation score and sleepiness level. For analyses involving sleep condition, individuals were excluded if their average TIB across both sleep restriction nights exceeded 5 h 30, or if their TIB exceeded 5 h 30 on the night before the lab visit. For the sleep saturation conditions, participants were excluded if their average TIB was less than 7 h 30 or less than 7 h the night before the lab visit. These cut‐offs were chosen to induce a substantial but ecologically relevant difference in sleep opportunity, while allowing for natural variability in sleep patterns that occurs in participants' home environments. As a result, 8/53 in the sleep saturation no‐vaccine condition, 24/50 in the sleep restriction no‐vaccine condition, 5/26 in the sleep saturation vaccine condition, and 9/30 in the sleep restriction vaccine condition were excluded from analyses involving sleep condition effects. See Results for the mean TIB and total sleep time (TST). The applied thresholds ensured that the difference in TIB between sleep conditions was a minimum of 4 h over the two nights. Actigraphy data were unavailable for six out of 159 sessions (3.8%); their MOSS data were included in the final analyses. Cytokine data were missing for one out of 159 sessions. Cytokine values exceeding four standard deviations from the mean were excluded, accounting for 1.4% of the data (13 out of 947 observations; one additional value was missing before cleaning).

## 3 Results

### 3.1 Time in Bed and Total Sleep Time in the Sleep Conditions

#### 3.1.1 Time in Bed

Across the two nights of sleep manipulation, participants' average TIB in the sleep saturation condition was 9 h01 ± 0 h32 (range: 7 h50–10 h30) in the no‐vaccine condition and 8 h50 ± 0 h44 (range: 7 h48–10 h17) in the vaccine condition (*b* = 11 m46, SE = 8 m38, *p* = 0.528). In the sleep restriction condition, the average TIB was 4 h32 ± 0 h27 (range: 3 h52–5 h30) in the no‐vaccine condition and 4 h25 ± 0 h30 (range: 3 h44–5 h27) in the vaccine condition (*b* = 7 m48, SE = 9 m29, *p* = 0.844). The average sleep restriction effect was 4 h29 (95% CI [−4 h44, −4 h13], *p* < 0.001).

#### 3.1.2 Total Sleep Time

Across the two nights of sleep manipulation, participants' average TST in the sleep saturation condition was 8 h02 ± 0 h38 (range: 6 h27–9 h45) in the no‐vaccine condition and 7 h55 ± 0 h48 (range: 6 h36–9 h45) in the vaccine condition (*b* = 10 m33, SE = 8 m24, *p* = 0.594). In the sleep restriction condition, the average TST was 4 h03 ± 0 h23 (range: 3 h11–4 h44) in the no‐vaccine condition and 3 h58 ± 0 h31 (range: 3 h01–5 h01) in the vaccine condition (*b* = −1 m36, SE = 9 m40, *p* = 0.999). The average sleep restriction effect was 4 h01 (95% CI [−4.16, −3.46], *p* < 0.001).

### 3.2 Sleep Restriction and Vaccine Effects on Sleepiness, Inflammation, and Body Temperature

#### 3.2.1 Sleepiness

Subjective sleepiness, assessed using the Karolinska Sleepiness Scale (KSS), showed a significant effect of sleep restriction (*b* = 2.98 scale steps, 95% CI [2.33, 3.63], *p* < 0.001). The main effect for vaccine and the vaccine × sleep interaction were not retained in the final model (Figure 2A).

**FIGURE 2:**
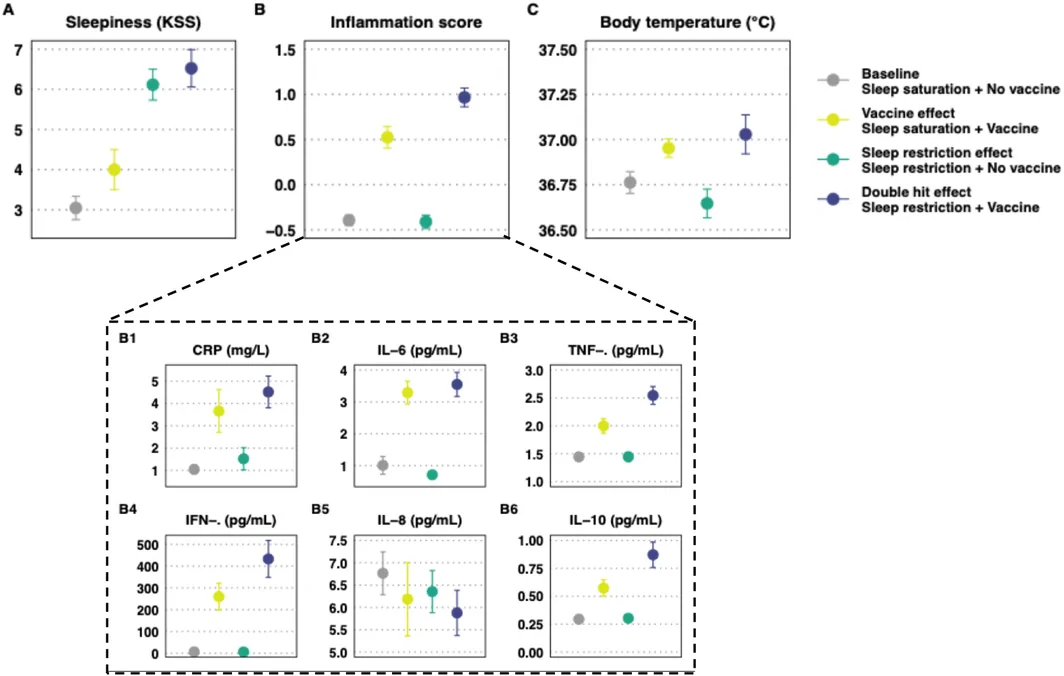
Mean levels of sleepiness (Karolinska Sleepiness Scale; KSS), inflammation score (cytokine composite) and body temperature (degrees Celsius; °C) for each experimental condition. The four conditions include: *Baseline* (sleep saturation + no vaccine, *n* = 45), *vaccine effect* (Sleep saturation + Vaccine; *n* = 21), *sleep restriction effect* (Sleep restriction + no vaccine, *n* = 26), and *double hit effect* (combination of sleep restriction and vaccine, *n* = 21). The plots display condition means with error bars representing standard errors. Subpanels B1–B6 show the raw levels of individual cytokine that were included in the composite inflammation score.

#### 3.2.2 Inflammation

A significant main effect of vaccine indicates higher inflammation levels after vaccination (*b* = 0.93, 95% CI [0.72, 1.14], *p* < 0.001). No significant main effect of sleep restriction was observed (*b* = −0.03, 95% CI [−0.21, 0.15], *p* = 0.742). A significant vaccine × sleep restriction interaction (*b* = 0.45, 95% CI [0.14, 0.77], *p* = 0.005) indicates that sleep loss amplified the inflammatory response to vaccination (Figure 2B).

#### 3.2.3 Body Temperature

Vaccination led to a small increase in body temperature (*b* = 0.24°C, 95% CI [0.13, 0.34], *p* < 0.001) (range 35.6°C–37.8°C). The main effect for sleep restriction and the vaccine × sleep restriction interaction were not retained in the final model (Figure 2C).

Sleepiness and the inflammation score were positively correlated, with each scale step increase on the KSS being associated with a 0.05‐point increase in the inflammation score (95% CI [0.00, 0.10], *p* = 0.035). Adding the participant's COVID‐19 vaccine history (whether receiving their 1st, 2nd, or 3rd COVID‐19 vaccination in this study), gender, BMI, or testing time (morning or afternoon) to the model did not alter any of the conclusions reported above. See Table S5 for detailed results of the adjusted models.

### 3.3 Effects of Vaccination and Sleep Restriction on Motivation

As shown in Figure 3 and Table 1, both vaccination and sleep restriction increased motivation to engage in sleep preparatory behaviours, resting and sleeping and decreased energy demanding activities. Specifically, a stronger desire to be at home, close one's eyes and be in a quiet room (Figure 3A), wanting to rest and sleep (Figure 3B), and wanting to be alone (Figure 3E), and decreased desire for physically active behaviours, such as walking and exercising (Figure 3D).

**FIGURE 3:**
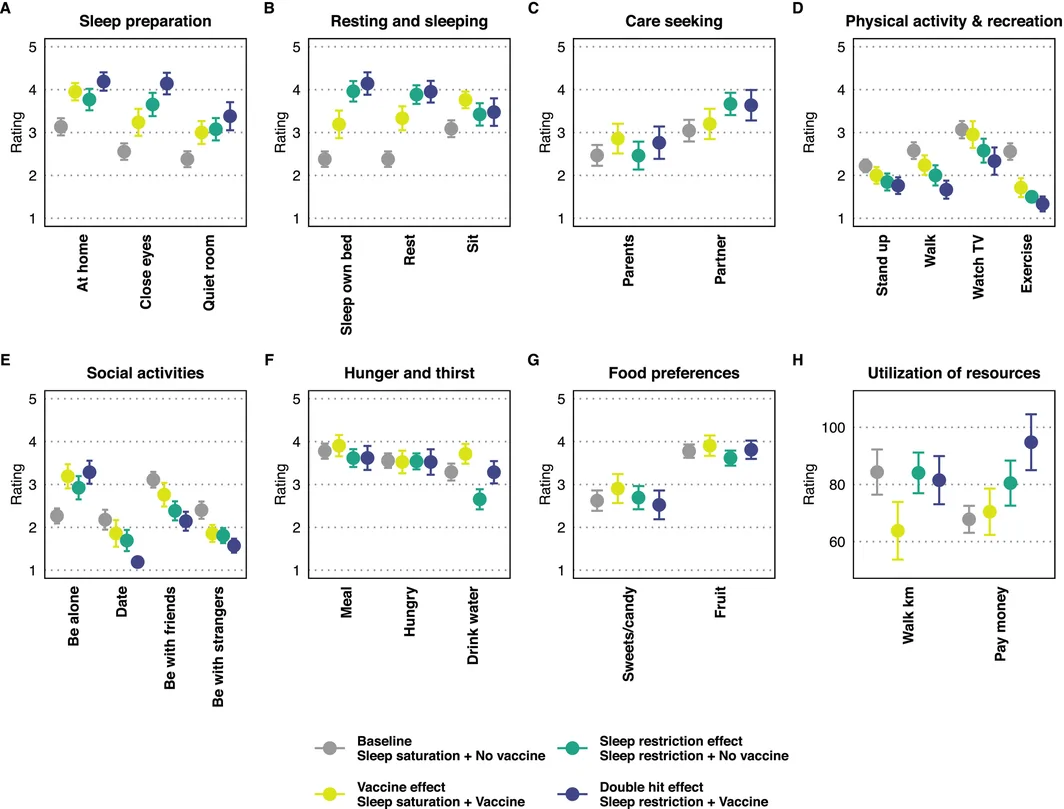
Mean levels of motivation items for each experimental condition. The four conditions include: *Baseline* (sleep saturation + no vaccine, *n* = 45), *vaccine effect* (Sleep saturation + Vaccine; *n* = 21), *sleep restriction effect* (Sleep restriction + no vaccine, *n* = 26), and *double hit effect* (combination of sleep restriction and vaccine, *n* = 21). The plots display condition means with standard error bars. See Table 1 for the corresponding coefficients and 95% confidence intervals.

**TABLE 1:** Results of the mixed effect models, displaying the coefficient (b), 95% confidence interval (CI), and *p* value for each motivation item with vaccine effect (coded 0 = no vaccine, 1 = vaccine), sleep restriction (coded 0 = sleep saturation, 1 = sleep restriction), and their interaction.

Category	Outcome	Vaccine effect			Sleep restriction effect			Vaccine × sleep restriction		
		*b*	95% CI	*p*	*b*	95% CI	*p*	*b*	95% CI	*p*
Sleep preparation	At home	0.49*	**0.12, 0.87**	**0.011**	0.70*	**0.31, 1.08**	**0.001**			
	Close eyes	0.47*	**0.07, 0.88**	**0.022**	1.15*	**0.74, 1.57**	**0.000**			
	Quiet room	0.43*	**0.02, 0.84**	**0.041**	0.49*	**0.07, 0.91**	**0.024**			
Resting and sleeping	Rest	0.42*	**0.06, 0.78**	**0.023**	1.26*	**0.88, 1.63**	**0.000**			
	Sleep own bed	0.42*	**0.03, 0.81**	**0.035**	1.40*	**1.00, 1.81**	**0.000**			
	Sit				0.20	−0.23, 0.63	0.359			
Care seeking	Parents				0.27	−0.08, 0.62	0.135			
	Partner				0.58	−0.19, 1.34	0.134			
Physical activities and recreation	Stand up				−0.33*	**−0.65, 0.00**	**0.049**			
	Walk	−0.37*	**−0.74, 0.00**	**0.048**	−0.53*	**−0.91, −0.16**	**0.006**			
	Exercise	−0.90*	**−1.4, −0.39**	**0.001**	−1.05*	**−1.51, −0.59**	**0.000**	0.77*	**0.00, 1.53**	**0.049**
	Watch TV				−0.63*	**−1.09, −0.17**	**0.007**			
Social activities	Be alone	0.57*	**0.19, 0.95**	**0.004**	0.51*	**0.12, 0.90**	**0.011**			
	Be with friends				−0.72*	**−1.14, −0.31**	**0.001**			
	Be with stranger	−0.37*	**−0.74, 0.00**	**0.049**	−0.50*	**−0.87, −0.12**	**0.010**			
	Blind date				−0.58*	**−0.99, −0.17**	**0.006**			
Hunger and thirst	Hungry				0.05	−0.33, 0.43	0.809			
	Drink water	0.51*	**0.06, 0.95**	**0.028**	−0.54*	**−0.99, −0.09**	**0.019**			
	Meal				−0.08	−0.43, 0.28	0.672			
Food preferences	Fruit				−0.06	−0.41, 0.29	0.733			
	Sweets/candy				0.02	−0.37, 0.42	0.903			
Utilisation of resources	Pay money (rank)				18.23*	**6.32, 30.13**	**0.003**			
	Walk km (rank)				1.39	−12.54, 15.31	0.843			
Physiological measures	Sleepiness				2.98*	**2.33, 3.63**	**0.000**			
	Inflammation score	0.93*	**0.72, 1.14**	**0.000**	−0.03	−0.21, 0.15	0.742	0.45*	**0.14, 0.77**	**0.005**
	Body temperature	0.24*	**0.13, 0.34**	**0.000**						

*Note:* Blank cells indicate that terms were removed during backward model selection. Asterisks (*) and bold denote statistically significant effects (*p* < 0.05).

In the vaccine condition, participants were more motivated to be alone and avoid socialising with strangers (Figure 3E), walking or exercising (Figure 3D), but other social (e.g., being with friends, going on a date) and physical activities (e.g., standing up, watching TV) (Figure 3D,E) were not significantly affected, as opposed to sleep restriction that reduced motivation for all types of social and physical activity. The vaccine × sleep condition interaction for exercise motivation indicated that vaccination significantly reduced motivation for exercise in the sleep saturation condition (*b* = 0.90, SE = 0.26, *p* = 0.007) but had no additional impact under sleep restriction (*b* = 0.13, SE = 0.28 *p* = 0.659). The preparedness to pay money in exchange for bedtime access (Figure 3H) increased significantly after sleep restriction, but not after vaccination, but participants were not willing to walk for bedtime access in either condition. Finally, wanting to drink water decreased following sleep restriction, but increased after vaccination. Neither vaccination nor sleep restriction affected hunger or food preferences (Figure 3F,G).

### 3.4 The Combined Effects of Vaccination and Sleep Restriction on Physiological Measures and Motivation

As shown in Figure 3 and Table 1, there was no support for the physiological challenges to amplify each other's effects for any of the measured motivation outcomes. Instead, the impact of vaccination and sleep restriction on motivation was additive in nature. The combined effect of both conditions was statistically greater than from vaccine alone for motivation for sleep‐preparatory behaviours (closing one's eyes, resting, sleeping in one's own bed), social activities (go on a blind date), resource utilisation (paying money in exchange for bedtime access), and physiological measures (body temperature and inflammation score). For the inflammation score, the combined effect was also statistically greater than the effect of sleep restriction alone. See Supplement for details of the contrast tests.

### 3.5 Relationships of Inflammation and Sleepiness Levels With Motivation

#### 3.5.1 Inflammation

As shown in Figure 4, higher inflammation scores were associated with increased motivation for sleep‐preparatory behaviours, resting and sleeping, and to be taken care of by one's parents (but not partner). The motivation for physical exercise and walking, but not sitting, standing or watching TV, decreased with a higher inflammation score. The desire to be alone increased and the desire to go on a blind date was lower with a higher inflammation score, but motivation to be with a stranger or friends was not significantly associated with the inflammation score. A higher inflammation score was also associated with greater preparedness to utilise monetary resources to be allowed to go to bed, but not with the preparedness to utilise energy resources (walking). Hunger, thirst and food preferences were not associated with inflammation scores. See also Table S7 for results.

**FIGURE 4:**
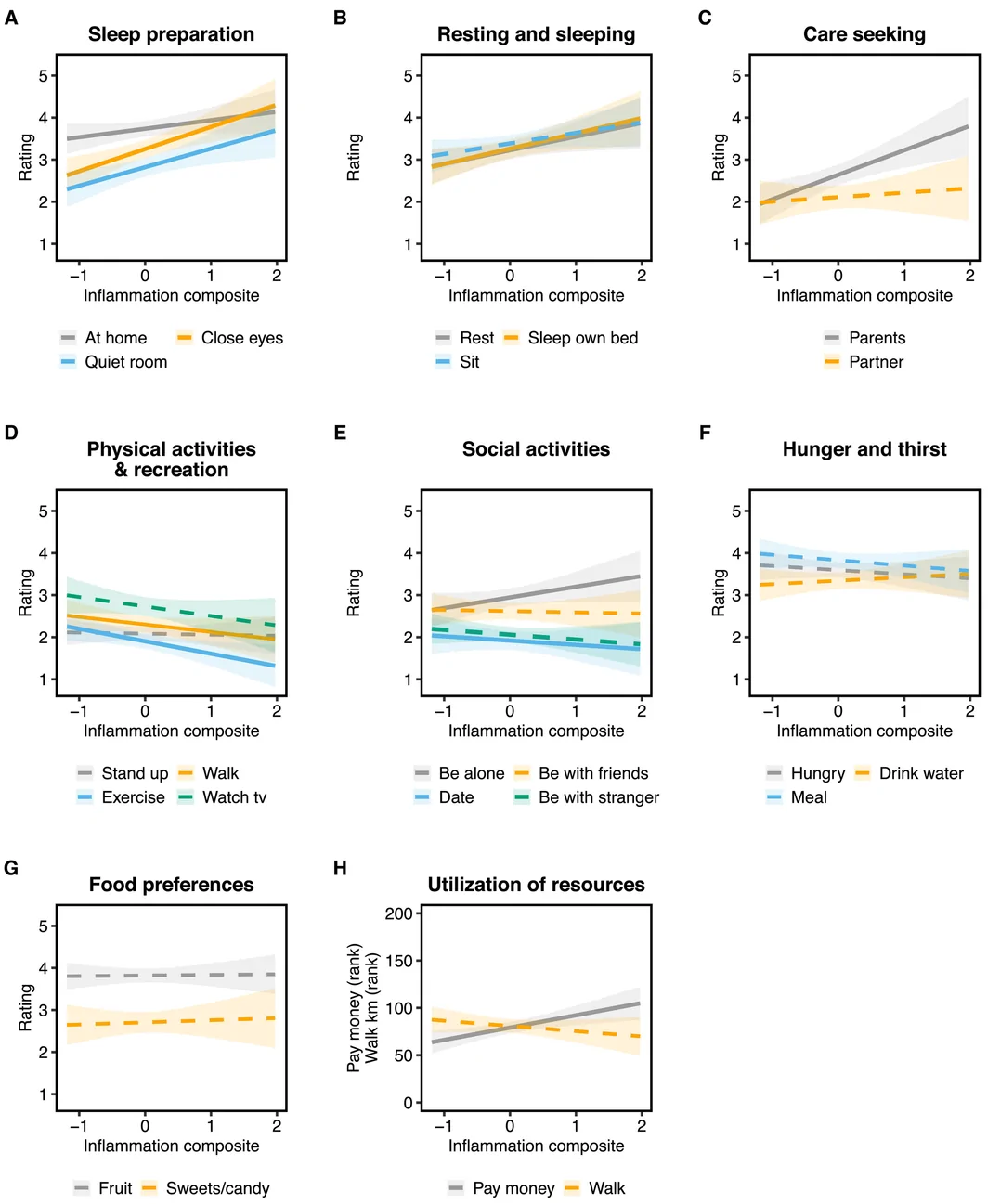
Relationship between inflammation scores (composite scores) and motivation items. Motivation items are presented as (A–G) means of the responses (1 = not at all, 5 = very much) and (H) preparedness to utilise monetary or energy resources to be allowed to go to bed, with responses being rank transformed. The solid lines indicate statistical significance (*p* < 0.05), and the dashed lines indicate non‐significance (*p* > 0.05) determined by linear mixed effects models. See Table S7 for the coefficients and corresponding 95% confidence intervals.

#### 3.5.2 Sleepiness Level

As shown in Figure 5, sleepiness was associated with increased motivation for sleep‐preparatory behaviours, resting and sleeping, and wanting to be taken care of by one's parents or partner. Motivation for all physical activities and recreation as well as motivation to be socially active with anyone decreased with higher sleepiness. Sleepiness level was also associated with a greater preparedness to utilise monetary resources for bedtime access, but not the preparedness to utilise energy resources (walking). Hunger, thirst and food preferences were not associated with sleepiness. See Table S7 for detailed results.

**FIGURE 5:**
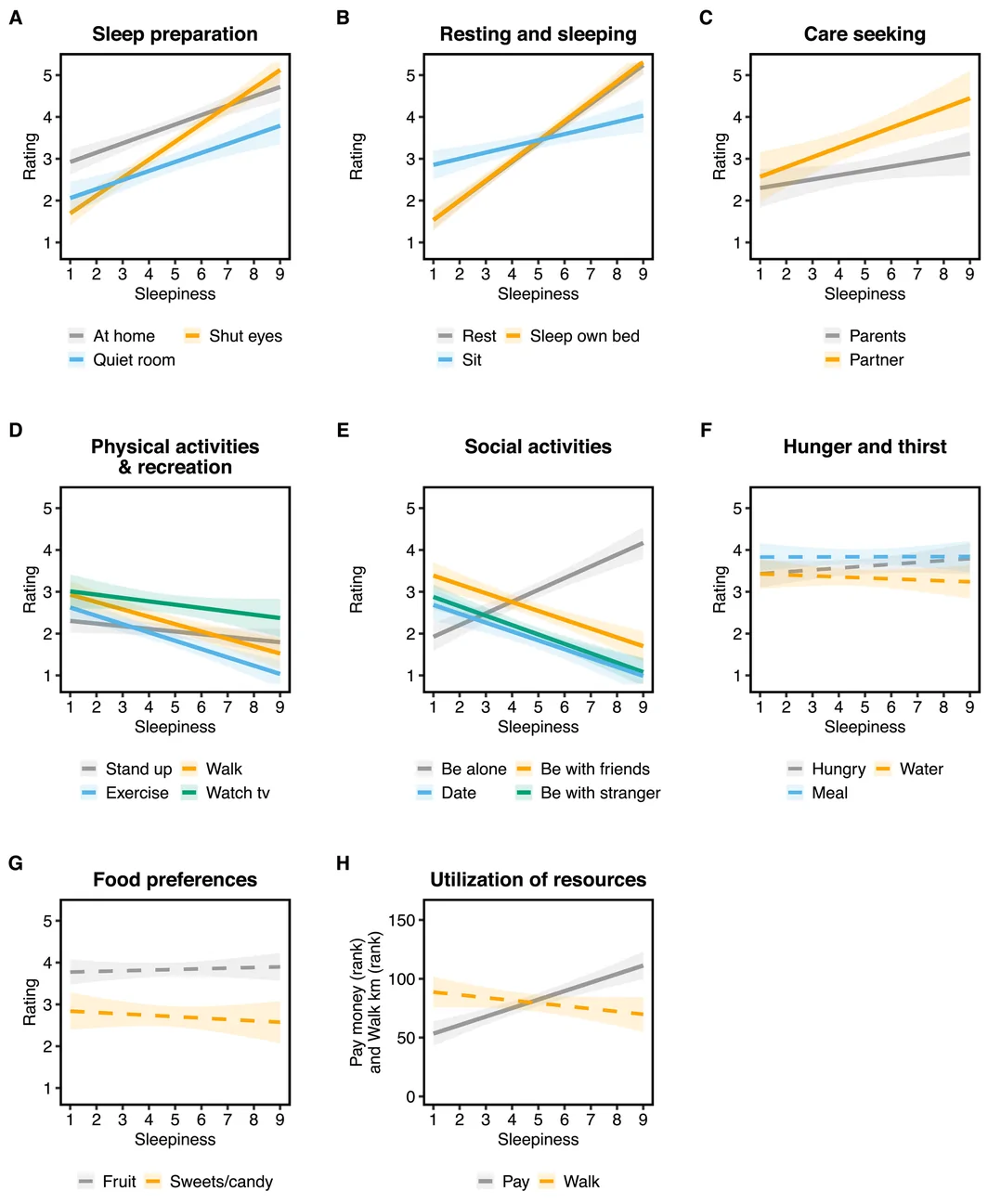
Relationship between sleepiness level (Karolinska Sleepiness Scale (KSS)) and motivation items. Motivation items are presented as (A–G) means of the responses (1 = very alert, 9 = very sleepy) and (H) preparedness to utilise monetary or energy resources to be allowed to go to bed, with responses being rank transformed. The solid lines indicate statistical significance (*p* < 0.05), and the dashed lines indicate non‐significance (*p* > 0.05) determined by linear mixed effects models. See Table S7 for the coefficients and corresponding 95% confidence intervals.

While both the inflammation score and sleepiness levels exhibit the same directional relationships with motivational factors, some differences emerge in their associations. Our analysis did not statistically compare the magnitude of these associations, but we compared the coefficients from the separate analyses of inflammation and sleepiness. The main distinction in their relationship with motivation lies in motivation for socialising with familiar people and for low physical activities, such as standing up or watching television. Individuals with higher sleepiness levels displayed lower motivation for these low physically active activities, correlations not found with inflammation. Both inflammation and sleepiness levels were associated with a greater desire to be alone and a lower motivation to go on a blind date. However, while higher sleepiness levels were associated with reduced motivation for socialising with friends, this was not the case for individuals with higher inflammation.

## 4 Discussion

Sleep restriction caused several motivational changes, with the strongest effects observed for sleep‐ and rest‐related behaviours (sleep in own bed, rest, close eyes), followed by a reduced desire for physical activity and social interaction with close others (exercise, be with friends), and increased desire for a safe environment (being at home). Sleep restriction also affected, in order of decreasing strength, the desire to watch TV, go on a blind date, drink water, walking, socialize with strangers, be cared for by a partner (increased), be alone, be in a quiet room and stand up. These findings extend work on total sleep deprivation (Axelsson et al. 2020) by demonstrating that also milder levels of sleep loss are sufficient to reorganise motivational priorities. Low‐grade inflammation triggered by COVID‐19 vaccination elicited broadly similar, though weaker, motivational changes, with strongest effects on desire to rest and to be alone, reduced desire for exercise, followed by increased desire for drinking water, and greater desire for sleep, rest, and be in a safe environment (at home, close eyes, quiet room, sleep in own bed). Thirst motivation diverged between the two stressors: sleep restriction decreased desire to drink water and vaccination increased it, possibly reflecting differing homeostatic pathways. Note that the magnitude of the inflammatory response or sleepiness level was not significantly correlated with the desire to drink water. Neither physiological stressor affected hunger or food preferences. Together, these findings demonstrate that both mild sleep loss and low‐grade inflammation shift motivational priorities towards conserving energy by promoting rest and solitude and reducing physical exertion. Yet, the specific patterns differ, with sleep loss broadly suppressing social and physical motivation, whereas inflammation targets more specific behaviours, such as avoidance of strangers and exercise, while sparing certain low‐effort and familiar social interaction.

Sleep loss amplified the inflammatory response to the COVID‐19 vaccine, indicating that insufficient sleep can prime the immune system to respond more strongly to an additional physiological challenge. Similar priming effects have been reported for other acute stressors at the time of antigen exposure (Dhabhar and Viswanathan 2005; Edwards et al. 2006; Dhabhar 2018). While stress‐induced increases in immune responses can improve immune‐protection during vaccination or infection, it may also contribute to prolonged inflammation‐induced sickness behaviour if not properly resolved. It is still unclear whether chronic conditions elicit synergistic effects on immune function. However, despite this immune amplification, the motivational effects of sleep loss and inflammation were additive in nature. Although the combined exposure was more detrimental than either physiological stressor alone for some outcomes, there was no evidence that the joint challenge amplified motivation effects. This pattern aligns with prior work showing linear increases in stress outcomes under multiple concurrent stressors. For instance, combinations of stressors, such as high body mass index (BMI) and aging, or sleep loss and social stress, show additive effects on outcomes such as inflammation, autonomic activity, subjective psychological stress, emotion recognition and psychomotor speed (Balter et al. 2021, 2019; Schwarz et al. 2018). A previous study found that pre‐existing sleep disturbances (habitual sleep) amplified depressed mood after experimental injections with endotoxin in women, but not men (Cho et al. 2016), and when sleep disturbances coincided with systemic inflammation it correlated with worse emotion recognition (Piber et al. 2020). Thus, different stressors may converge on similar motivational endpoints, they do not necessarily exacerbate each other's effects beyond additivity, at least in acute experimental contexts. While the experimental induction of the physiological stressors allowed us to isolate their immediate impact in the present study, they were both acute in nature, and it is plausible that the effects might be amplified in protracted states such as chronic sleep loss or sustained inflammatory states. Clearly, there is a need to increase the knowledge on when stressors act in an additive and when multiplicative manner.

No changes in hunger or food preferences were observed following inflammation, contradicting some previous studies on inflammation (Chareh et al. 2021; Lee et al. 2007; Nordgreen et al. 2018; Vichaya et al. 2014), although these studies of inflammation focused on more potent inflammation and correlational studies, mostly in older adults. That sleep restriction did not robustly impact hunger or food preferences is in line with a previous study on total sleep deprivation (Axelsson et al. 2020), but contrasts studies showing that sleep deprivation increases hunger and preference for high‐energy foods (Zhu et al. 2019; Markwald et al. 2013). There is also evidence from animal studies that REM‐deprivation reduces food intake (Hanlon et al. 2005), suggesting that the influence of insufficient sleep on hunger and food preferences is multifaceted, likely influenced by metabolic status, learnt behaviour and contextual factors. Our null findings suggest that altered hunger and food preferences may play a stronger role during more severe physiological stress and/or at later stages of recovery. These alterations could serve as a functional response to maintain homeostasis when faced with metabolic challenges and prolonged exposure to stress, such as seen in individuals exposed to 5 days of insufficient sleep (Markwald et al. 2013). Nevertheless, wanting to drink water increased after vaccination and decreased after sleep restriction, possibly reflecting distinct homeostatic pathways such as thermoregulation as a result of vaccine‐induced elevations in body temperature. Alterations in hunger and food preferences may not be central to the initial recovery process following acute allostatic challenges, but could represent secondary responses that emerge once sleep and initial recovery have occurred.

While sleep loss and vaccine‐induced inflammation produced similar motivational changes, the mechanistic underpinnings likely differ. Peripheral inflammation and subjective sleepiness were both associated with motivational changes. The primary difference between peripheral inflammation and sleepiness levels in their relationship with motivation emerged for social motivation for interacting with familiar people and low‐effort physical activities, which were negatively associated with sleepiness but not with inflammation levels. This suggests that social motivation towards close others is preserved during low‐grade inflammation, in contrast to the heightened social withdrawal observed during more severe experimental sickness (Inagaki et al. 2015). At the neural level, the ventral tegmental area (VTA), a group of midbrain neurons central for the mesolimbic dopamine system and motivation (Treadway et al. 2019), has been implicated in inflammation‐induced motivation and behavioural changes, and recent findings suggest that other stressors can elicit similar responses. For example, social defeat stress in mice increased sleep need via VTA neurons inhibiting corticotropin releasing hormone (CRH), which allowed these mice to sleep for longer (Yu et al. 2022). It thus seems that the VTA serves as a hub where different stress signals can trigger a general recovery response, or at least have significant overlap. While there is a growing understanding of how peripheral immune responses affect the brain (Dantzer et al. 2008; Thylur and Goldsmith 2022), we have a poorer understanding of the mechanistic drivers of sleep homeostasis. Recent work suggests that calcium changes play a central role in the buildup of sleep pressure and sleep loss (Ingiosi et al. 2020; Wang et al. 2022).

Considering that both low‐grade inflammation and sleepiness are common in various disorders and predict disease progression (Ernst et al. 2021; Eswarappa et al. 2019), the relevance of these findings extends to multiple domains. Reduced motivation may represent a mechanistic link between stress exposure and health behaviour change, potentially increasing risk over time. Therapeutic interventions that target both sleep and inflammation may therefore have downstream benefits for motivation and behaviour in vulnerable populations.

Several limitations of the current study should be noted. The absence of a placebo injection (as opposed to no injection) may have introduced noise via a potential nocebo effect (Haas et al. 2022). Additionally, there was variability in the participants' vaccination histories, though statistical adjustment of this did not impact the results. While a seven‐day washout period was implemented between sessions, the possibility of residual effects cannot be fully excluded. Also, the many analyses carried out also increase the risk of Type‐I errors; however, while a few of the significant effects may be random, the consistent and clear patterns observed for both physiological stressors, along with the close replication of motivational changes seen in a previous study involving more severe sleep deprivation (Axelsson et al. 2020), support the robustness of the findings. Adherence to the sleep restriction protocol was variable and lower than typically observed in controlled laboratory settings. Conducting the protocol in participants' home environments increased ecological validity but also introduced greater variability in compliance. Self‐selection effects cannot be ruled out; individuals who tolerate sleep restriction more easily may have been more likely to adhere to the protocol, potentially biassing estimates of sleep loss effects towards the null. Consequently, the observed effects may represent conservative estimates, and the true impact of sleep loss, and its combined effect with inflammation on motivation, may be larger than reported. Strengths include experimental manipulation of sleep loss and low‐grade inflammation and within‐subject assessments allowing comparison of the vaccine condition to both sleep saturation and sleep restriction. The extent of motivational changes is likely influenced by an individual's need for sleep, tolerance to sleep loss, and the amount of sleep loss or disturbed sleep, as well as contextual factors, aspects that warrant further investigation. There is also a need for further research to replicate our findings and to study how other and more chronic stressors influence motivation, and their translation to wellbeing, behaviour, and health risks. It is important to note that vaccination had generally weaker effects. Generalizability of the findings also needs to be challenged by studies in other research groups and using alternative (physiological) stressors. The Motivational Scale of Sleepiness would also benefit from psychometric validation, and future studies could incorporate multiple blood sampling timepoints per session to further characterise the inflammatory kinetics of COVID‐19 vaccination as a novel experimental model of low‐grade inflammation. Future research could also explore breaking down the composite inflammatory score into subcategories to test whether different inflammatory profiles relate to distinct motivational changes. This may offer more insights into the immune–motivation link, although such approaches would require careful theoretical justification and adequate sample sizes to retain statistical power. We will also share our data so that others can carry out additional analyses and combine data from several data collections.

To conclude, both partial sleep loss and a mild immune stressor induce a coordinated motivational response that prioritises rest, safety, and withdrawal from physically and socially demanding activities without altering hunger and food preferences, with the likely purpose to support homeostasis and recovery. These two physiological stressors induced similar motivational changes although they differ in magnitude, and they may operate through partly different mechanisms. The primary difference was that motivation for being with familiar people and motivation for lower effort physical activities were maintained after inflammation and with a stronger inflammatory response. Given the high societal prevalence of sleep loss, sleepiness, and low‐grade inflammation, these modifiable states represent potential research and therapeutic targets for alleviating motivational deficits in states associated with inflammation and sleep loss.

## Author Contributions

Conceptualization: John Axelsson and Leonie J.T. Balter. Investigation: Leonie J.T. Balter. Data curation: Leonie J.T. Balter. Formal analysis: Leonie J.T. Balter. Cytokine and Chemokine analysis: Camilla I. Svensson, Nils Simon, Alexandra Kuliszkiewicz and Leonie J.T. Balter. Visualisation and figures: Leonie J.T. Balter. Methodology: John Axelsson and Leonie J.T. Balter. Writing‐original draft: John Axelsson and Leonie J.T. Balter. Writing‐review and editing: John Axelsson, Mats Lekander, Camilla I. Svensson, Nils Simon, Alexandra Kuliszkiewicz, Julie Lasselin and Leonie J.T. Balter. Funding acquisition: John Axelsson and Leonie J.T. Balter.

## Funding

This study was supported by grants from Riksbankens Jubileumsfond (Swedish Foundation for Humanities and Social Sciences (grant no. P13‐1159:1) [to John Axelsson]), Lisa och Johan Grönbergs Stiftelse (grant no. 2022–00218) [to Leonie J.T. Balter], Karolinska Institutet Research(grant no. 2022–01832) [to Leonie J.T. Balter], European Union's Horizon 2020 research and innovation programme under the Marie Skłodowska‐Curie grant no 101108620 [to Leonie J.T. Balter], the Knut and Alice Wallenberg Foundation [to Camilla I. Svensson], the European Research Council (ERC) under the European Union's Horizon 2020 Research and Innovation Programme (grant agreement no. 866075) [to Camilla I. Svensson], and the European Union Horizon 2020 research and innovation program under the Marie Sklodowska‐Curie grant agreement no. 814244 (BonePain II) [to Nils Simon].

## Ethics Statement

This study was approved by the Swedish Ethical Review Authority (dnr: 2021–03730).

## Conflicts of Interest

The authors declare no conflicts of interest.

## Supporting information


**Data S1:** jsr70335‐sup‐0001‐Supinfo.pdf.

## Data Availability

The data that support the findings of this study are available in OSF at https://doi.org/10.17605/OSF.IO/ET7V5.
