# Supplementary material for: Peripheral Inflammation and Sleep Loss Induce Coordinated Motivational Changes: An Experimental Two‐Hit Stress Model
Source: J Sleep Res. 2026 Apr 27;35(5):e70335. doi: 10.1111/jsr.70335 (PMC13619025; doi:10.1111/jsr.70335)
Supplement: Supplementary file 1 [file JSR-35-e70335-s001.pdf]

Supplementary information for:

Peripheral inflammation and sleep loss induce coordinated motivational changes: An experimental two-hit stress model

Table of Contents

Supplementary methods **3**

Rationale for using the experimental manipulations of choice *3*

MOSS modifications *3*

Additional contrast tests *4*

Supplementary figures **5**

Figure S1. Predictors retained in final models testing the effect of vaccine condition, sleep condition, and their interaction *5*

Supplementary Tables **6**

Table S1. Overview of the COVID-19 vaccine history *6*

Table S2. Items of the motivation scale of sleepiness (MOSS). *7*

Table S3. Model comparisons of mixed effect structures for body temperature, inflammation composite, and sleepiness *8*

Table S4. Model comparisons of mixed effect structures for the motivation items *9*

Table S5. Adjusted mixed-effects models for each motivation item and physiological measure *11*

Table S6. Condition effects versus baseline (no vaccine + sleep saturation) *13*

Table S7. Results of the mixed effect models for Figure 4 and Figure 5 *15*

Table S8. Results of contrast tests examining the combined effects of vaccination and sleep restriction across all outcome variables *16*

SI References **18**

Supplementary methods

Rationale for using the experimental manipulations of choice

Experimental models of acute inflammation and sleep loss have provided insights into the mechanisms underlying cognitive, emotional, and behavioural alterations induced by these factors [1,2]. Experimental sleep loss designs typically involve full sleep deprivation, entailing a complete absence of sleep for at least one night, or partial sleep restriction, which can involve disrupted and/or shortened sleep duration over one night or longer [3–6]. Acute inflammation studies commonly administer an immune activator such as *Salmonella Typhi* or an influenza vaccine to induce low-grade inflammation [7–9] or lipopolysaccharide (LPS) for potent inflammation levels [10,11]. In the present study, we used partial sleep restriction for two nights (instructed time in bed 4 h/night). This duration was chosen as an ecologically valid method to mimic a common pattern of sleep disruption experienced by many individuals in real-world settings. We combined partial sleep restriction with a novel model of acute inflammation, namely COVID-19 vaccination. This vaccination, shown to acutely induce a peripheral immune response [12], has thus far seen limited application in studying the effects of inflammation on cognition and behaviour.

MOSS modifications

For the current study, we made slight modifications to the MOSS compared to the version used in [13]. Specifically, three items were added: wanting to stand up; wanting to sit down; and wanting to watch TV/film/series (or something similar); and three items were removed: wanting to lie down in the nearest possible bed (and sleep); wanting to go grocery shopping; and wanting to eat a juicy steak (or other protein-rich food).

Additional contrast tests

To assess whether the combined effect of vaccination and sleep restriction on each outcome was additive or interactive, estimated marginal means (EMMs) were computed for all four condition combinations (vaccine condition = 0/1 × sleep condition = 0/1). We then conducted a contrast test of additivity, defined as: D (combined effect) – B (vaccine only) – C (sleep restriction only) + A (baseline) = 0, with the null hypothesis supporting additivity indicating that the combined effect does not significantly differ from the sum of the individual effects. To further test whether the combined condition (D) produced a significantly greater effect than either challenge alone, we also compared D vs. B (vaccine effect) and D vs. C (sleep restriction effect) directly. These tests assessed whether the joint challenge produced a larger effect than vaccine alone and sleep restriction alone, respectively. Results of all contrasts are reported in the Table S8. The main findings were that for most outcomes, the joint effect of vaccine and sleep restriction did not exceed the sum of the individual effects, suggesting no interactive effect. However, the combined challenge produced stronger effects than vaccine alone for wanting to close one's eyes, rest, sleep, willingness to use financial resources for bedtime access, as well as sleepiness and inflammation. The combined challenge rarely exceeded those of sleep restriction alone, suggesting that sleep loss accounted for most of the observed effects.

Supplementary figures

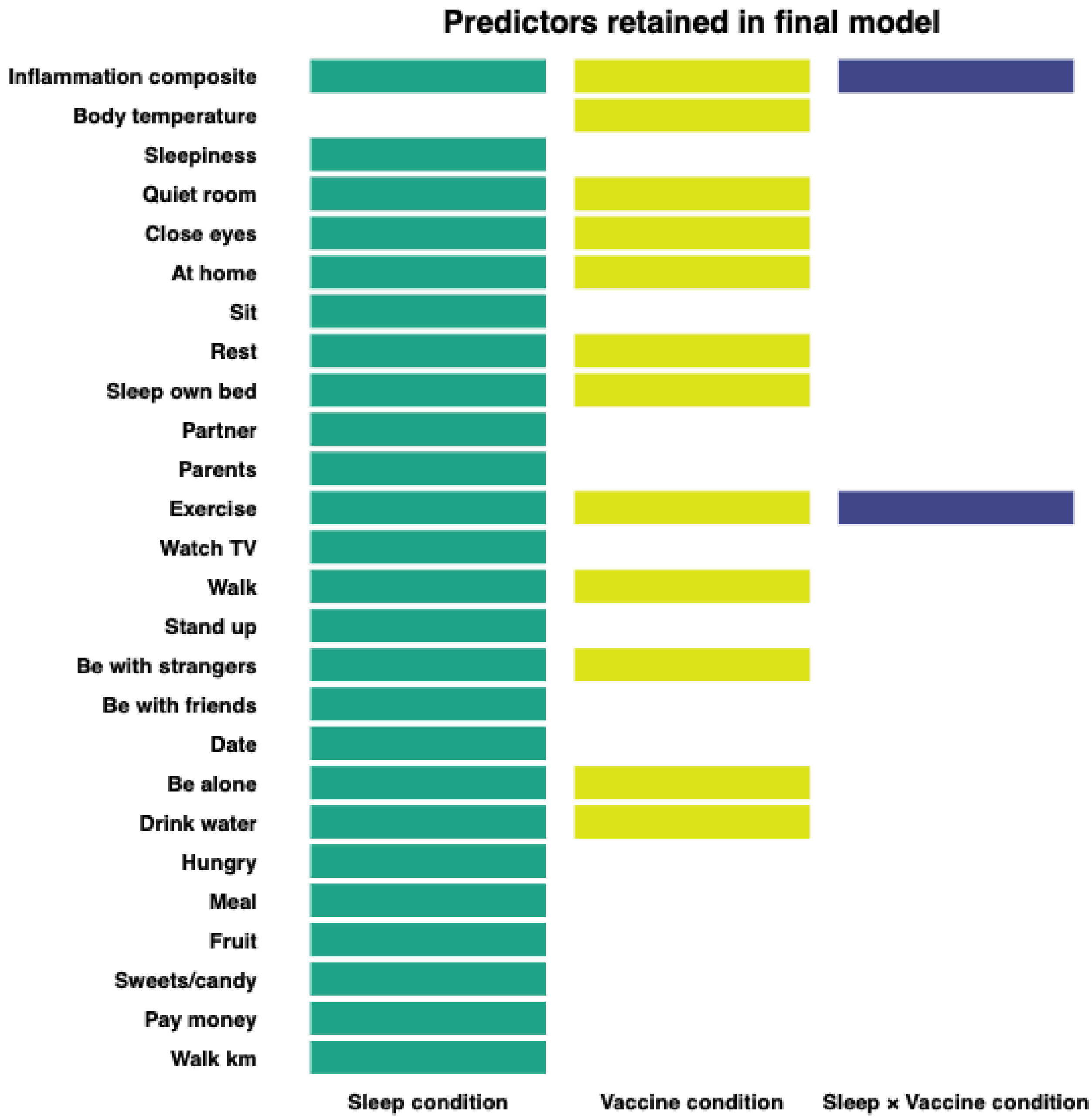

Figure S1. Predictors retained in final models testing the effect of vaccine condition, sleep condition, and their interaction. Coloured tiles indicate which predictors were retained in the final model for each item after backward model selection using the *Buildmer* R package [14]. Predictors include *Sleep condition*, *Vaccine condition*, and their interaction (*Vaccine* × *Sleep condition*). The random intercept for participant (ID) was included in all models and was not subject to removal during model selection. The inflammation composite and one MOSS item ("Exercise") retained the interaction term.

Supplementary Tables

Table S1. Overview of the COVID-19 vaccine history and the COVID-19 vaccines participants received in the study

Brand	COVID-19 history (prior to study)		Vaccination received in the study
	First dose	Second dose	
AstraZeneca	4	3	0
Johnson & Johnson	2	0	0
Moderna	5	2	3
Pfizer	37	7	53
Sinopharm	4	2	0
Info missing	1	1	0

Note. Three participants received their first vaccine dose as part of the current study, 38 their second vaccine dose, and 15 their third vaccine dose.

Table S2. Items of the motivation scale of sleepiness (MOSS).

All responses are on the scale of 1 = not at all - 5 = very much, unless otherwise indicated
1. How hungry are you right now?
2. How much do you want to rest right now?
3. How much do you want to lie down in your own bed (and sleep) right now?
4. How much do you want to stand up right now?
5. How much do you want to go for a walk right now?
6. How much do you want to work out right now?
7. How much do you want to be at home right now?
8. How much do you want to be alone right now?
9. How much do you want to be with friends right now?
10. How much do you want to socialize with strangers right now?
11. How much would you like to go on a date with a stranger right now?
12. How much would you like to drink a glass of water right now?
13. How much would you like to sit down right now?
14. How much would you like to have a meal right now?
15. How much would you like to have a fruit right now?
16. How much would you like to have candy (or other sweets) right now?
17. How much would you like to watch TV/film/series (or something similar) right now?
18. How much do you want to close your eyes right now?
19. How much do you want to be taken care of by your partner right now? (0 = I don't have a partner)
20. How much do you want to be taken care of by a parent right now?
21. How much do you wish it was completely quiet in the room you are in right now?
22. How much would you be willing to pay to go to bed right now? (0, 2, 5, 10, 50 euro, other amount: __)
23. If the alternative was staying awake for another 24 hours from now, how many kilometres would you be prepared to walk if you could go to bed right afterwards? Please indicate the maximum you would be willing to walk (it takes about 1 hour to walk 4 kilometres). (0, 1, 2, 4, 8, 12, other distance:___ kilometres)

Table S3. Model comparisons of mixed-effect structures for body temperature, inflammation composite, and sleepiness**.** Two models were evaluated: (1) a random intercept model *dv ~ vaccine condition * sleep condition* + *(1* | *ID)* and (2) a model including a random intercept and a random slope for sleep condition *dv ~ vaccine condition * sleep condition* + *(sleep condition* | *ID)*. Likelihood ratio tests were performed to evaluate whether adding a random slope for sleep condition improved model fit.

Outcome	***Model***	***AIC***	***BIC***	***Chisq***	***Df***	***Pr(>Chisq)***
Body Temperature	**intercept only**	110.66	127.02	NA		NA
Body Temperature	intercept + slope	115.4	139.95	1.26	3	0.740
Inflammation composite	**intercept only**	131.46	147.77	NA		NA
Inflammation composite	intercept + slope	137.45	161.92	0.00	3	1
Sleepiness	**intercept only**	476.38	492.74	NA		NA
Sleepiness	intercept + slope	480.17	504.71	2.21	3	0.530

Note. Bold highlighted models indicate better fit (p < 0.05) based on the likelihood ratio tests.

Table S4. Model comparisons of mixed-effect structures for the motivation items**.** Two models were evaluated: (1) a random intercept model *dv ~ vaccine condition * sleep condition* + *(1 | ID)* and (2) a model including a random intercept and an uncorrelated random slope for sleep condition due to convergence issues *dv ~ vaccine condition * sleep condition* + *(sleep condition* || *ID)*. Likelihood ratio tests were performed to evaluate whether adding a random slope for sleep condition improved model fit.

Outcome	*Model*	*AIC*	*BIC*	*Chisq*	*Df*	*Pr(>Chisq)*
At home	**intercept only**	**356.98**	**373.34**	**NA**		**NA**
At home	intercept + slope	362.91	387.46	0.06	3	0.996
Close eyes	**intercept only**	**376.73**	**393.1**	**NA**		**NA**
Close eyes	intercept + slope	382.73	407.28	0.00	3	1.000
Quiet room	**intercept only**	**376.06**	**392.42**	**NA**		**NA**
Quiet room	intercept + slope	379.9	404.45	2.15	3	0.541
Rest	**intercept only**	**351.08**	**367.44**	**NA**		**NA**
Rest	intercept + slope	355.78	380.33	1.3	3	0.730
Sleep own bed	**intercept only**	**366.06**	**382.42**	**NA**		**NA**
Sleep own bed	intercept + slope	372	396.54	0.06	3	0.996
Sit	**intercept only**	**374.17**	**390.53**	**NA**		**NA**
Sit	intercept + slope	377.81	402.35	2.36	3	0.501
Parents	**intercept only**	**385.04**	**401.4**	**NA**		**NA**
Parents	intercept + slope	390.74	415.29	0.30	3	0.960
Partner	**intercept only**	**NA**	**NA**	**NA**		**NA**
Partner	intercept + slope	NA	NA	NA		NA
Stand up	**intercept only**	**312.67**	**329.03**	**NA**		**NA**
Stand up	intercept + slope	318.59	343.14	0.08	3	0.994
Walk	**intercept only**	**352.57**	**368.93**	**NA**		**NA**
Walk	intercept + slope	358.47	383.02	0.10	3	0.992
Exercise	**intercept only**	**332.53**	**348.89**	**NA**		**NA**
Exercise	intercept + slope	330.73	355.28	7.80	3	0.050
Watch TV	**intercept only**	**394.4**	**410.76**	**NA**		**NA**
Watch TV	intercept + slope	392.94	417.49	7.46	3	0.059
Be alone	**intercept only**	**364.29**	**380.66**	**NA**		**NA**
Be alone	intercept + slope	368.25	392.79	2.05	3	0.562
Be with friends	**intercept only**	**360.9**	**377.27**	**NA**		**NA**
Be with friends	intercept + slope	366.9	391.44	0.00	3	1.000
Be with strangers	**intercept only**	**340.83**	**357.2**	**NA**		**NA**
Be with strangers	intercept + slope	342.19	366.73	4.65	3	0.200
Blind date	**intercept only**	**375.69**	**392.06**	**NA**		**NA**
Blind date	intercept + slope	375.08	399.63	6.61	3	0.085

Hungry	**intercept only**	**352.37**	**368.74**	**NA**		**NA**
Hungry	intercept + slope	358.37	382.92	0.00	3	1
Drink water	**intercept only**	**372.78**	**389.14**	**NA**		**NA**
Drink water	intercept + slope	378.66	403.21	0.12	3	0.989
Meal	**intercept only**	**347.83**	**364.19**	**NA**		**NA**
Meal	intercept + slope	353.82	378.37	0.01	3	1
Fruit	**intercept only**	**326.94**	**343.3**	**NA**		**NA**
Fruit	intercept + slope	331.5	356.05	1.43	3	0.697
Sweets/candy	**intercept only**	**391.84**	**408.2**	**NA**		**NA**
Sweets/candy	intercept + slope	396.68	421.23	1.16	3	0.763
Pay money	**intercept only**	**1131.88**	**1148.25**	**NA**		**NA**
Pay money	intercept + slope	1134.01	1158.55	3.87	3	0.275
Walk km	**intercept only**	**1172.99**	**1189.35**	**NA**		**NA**
Walk km	intercept + slope	1173.54	1198.09	5.45	3	0.142

Table S5. Adjusted mixed-effects models for each motivation item and physiological measure**,** with unstandardised coefficients (b), 95% confidence intervals (CI), and p-values. All models included gender (coded 0 = woman, 1 = man), BMI (continuous), vaccine dose (continuous), and time of testing (coded 0 = morning, 1 = afternoon), and a random intercept for participant ID. Fixed effects were selected using backward elimination, retaining only predictors that significantly improved model fit based on likelihood ratio tests. Asterisks (*) and bold font indicate statistically significant effects ($p < 0.05$). Only retained predictors are shown. Vaccine condition is coded as 0 = no vaccine, 1 = vaccine; sleep condition is coded as 0 = sleep saturation, 1 = sleep restriction.

Category	Outcome	Predictor	b	95% CI	p-value
Sleep preparation	At home	**Vaccine***	**0.47**	**0.10, 0.85**	**0.015**
		**Sleep***	**0.66**	**0.28, 1.04**	**0.001**
		**Gender***	**-0.62**	**-1.14, -0.10**	**0.024**
	Close eyes	**Vaccine***	**0.47**	**0.08, 0.87**	**0.022**
		**Sleep***	**1.15**	**0.74, 1.56**	**0.000**
	Quiet room	**Vaccine***	**0.46**	**0.06, 0.86**	**0.029**
		**Sleep***	**0.49**	**0.08, 0.90**	**0.023**
		**Vaccine dose***	**0.70**	**0.17, 1.22**	**0.012**
Resting and sleeping	Rest	**Vaccine***	**0.75**	**0.25, 1.25**	**0.004**
		**Sleep***	**1.49**	**1.04, 1.93**	**0.000**
		**Gender***	**-0.66**	**-1.20, -0.13**	**0.018**
		Vaccine x Sleep	-0.76	-1.53, 0.00	0.054
	Sleep own bed	**Vaccine***	**0.41**	**0.03, 0.79**	**0.038**
		**Sleep***	**1.40**	**1.01, 1.80**	**0.000**
		**Time of testing***	**-0.66**	**-1.22, -0.09**	**0.026**
	Sit	Sleep	0.20	-0.22, 0.62	0.359
Care seeking	Parents	Sleep	0.27	-0.07, 0.61	0.122
		BMI	0.06	-0.02, 0.14	0.155
		**Time of testing***	**1.14**	**0.35, 1.93**	**0.006**
	Partner	Sleep	0.51	-0.19, 1.22	0.160
		**Gender***	**-1.48**	**-2.32, -0.63**	**0.002**
		BMI	0.08	0.00, 0.16	0.066
Physical activities and recreation	Stand up	**Sleep***	**-0.33**	**-0.64, -0.01**	**0.049**
	Walk	**Vaccine***	**-0.37**	**-0.73, -0.01**	**0.048**
		**Sleep***	**-0.53**	**-0.91, -0.16**	**0.006**
	Exercise	**Vaccine***	**-0.57**	**-0.93, -0.20**	**0.003**
		**Sleep***	**-0.77**	**-1.14, -0.40**	**0.000**
		**Vaccine dose***	**-0.45**	**-0.84, -0.05**	**0.032**
	Watch TV	**Sleep***	**-0.63**	**-1.08, -0.18**	**0.007**
Social activities	Be alone	**Vaccine***	**0.58**	**0.21, 0.95**	**0.003**
		**Sleep***	**0.51**	**0.13, 0.90**	**0.011**
		BMI	0.05	-0.01, 0.11	0.086
		**Time of testing***	**-0.65**	**-1.23, -0.08**	**0.030**
	Be with friends	**Sleep***	**-0.72**	**-1.12, -0.32**	**0.001**
		**Time of testing***	**0.61**	**0.13, 1.09**	**0.016**
	Be with stranger	**Sleep***	**-0.53**	**-0.89, -0.16**	**0.006**
		**Gender***	**0.56**	**0.10, 1.01**	**0.021**
	Blind date	**Sleep***	**-0.54**	**-0.93, -0.14**	**0.009**

		**Gender***	**1.08**	**0.52, 1.65**	**0.000**
Hunger and thirst	Hungry	Sleep	0.04	-0.33, 0.42	0.814
		Time of testing	0.51	0.01, 1.01	0.053
	Drink water	**Vaccine***	**0.51**	**0.06, 0.95**	**0.028**
		**Sleep***	**-0.54**	**-0.98, -0.10**	**0.019**
	Meal	Sleep	-0.08	-0.43, 0.27	0.652
		**Time of testing***	**0.55**	**0.02, 1.08**	**0.046**
Food preferences	Fruit	Sleep	-0.06	-0.41, 0.29	0.733
	Sweets/candy	Sleep	0.02	-0.37, 0.41	0.916
		**Time of testing***	**0.80**	**0.06, 1.53**	**0.038**
Utilization of resources	Pay money (rank)	**Sleep***	**18.23**	**6.41, 30.06**	**0.003**
		BMI	0.55	-1.26, 2.36	0.554
	Walk km (rank)	Sleep	0.69	-13.00, 14.39	0.921
		**BMI***	**-3.16**	**-5.32, -1.01**	**0.006**
Physiological measures	Sleepiness	**Sleep***	**3.03**	**2.39, 3.66**	**0.000**
		BMI	0.09	-0.01, 0.19	0.096
	Inflammation score	**Vaccine***	**0.93**	**0.73, 1.13**	**0.000**
		Sleep	-0.03	-0.21, 0.15	0.742
		**Vaccine x Sleep***	**0.45**	**0.15, 0.76**	**0.005**
	Body temperature	**Vaccine***	**0.24**	**0.14, 0.34**	**0.000**
		**Time of testing***	**0.17**	**0.02, 0.33**	**0.033**

Table S6. Condition effects versus baseline (no vaccine + sleep saturation). Unstandardised coefficients (b) and corresponding 95% confidence intervals (CI) for each motivation item shown for the *vaccine effect*, *sleep restriction effect*, and *double hit effect*. The reference condition is No vaccine + sleep saturation (baseline) for all analyses. Asterisks (*) and bold denote statistically significant effects ($p < 0.05$). Utilization of resources relate to how much they would pay or walk (distance) to be allowed to sleep (rank transformed values). All other items were rated on a scale 1-5 (1=not at all, 5=very much).

**Category**	**Outcome**	**Vaccine effect** (Vaccine + sleep saturation vs baseline)		**Sleep effect** (No vaccine + sleep restriction vs baseline)		**Double hit effect** (Vaccine + sleep restriction vs baseline)	
		b	95% CI	b	95% CI	b	95% CI
Sleep preparation	At home	**0.55***	**0.01, 1.08**	**0.73***	**0.25, 1.21**	**1.17***	**0.64, 1.69**
	Close eyes	0.45	-0.12, 1.03	**1.14***	**0.62, 1.65**	**1.63***	**1.08, 2.19**
	Quiet room	0.55	-0.03, 1.13	**0.58***	**0.05, 1.10**	**0.87***	**0.30, 1.43**
Resting and sleeping	Rest	**0.77***	**0.26, 1.28**	**1.50***	**1.05, 1.96**	**1.53***	**1.03, 2.03**
	Sleep own bed	**0.69***	**0.14, 1.24**	**1.60***	**1.11, 2.10**	**1.71***	**1.17, 2.24**
	Sit	0.57	-0.02, 1.16	0.28	-0.25, 0.82	0.43	-0.15, 1.01
Care seeking	Parents	0.13	-0.38, 0.63	0.28	-0.16, 0.72	0.31	-0.17, 0.79
	Partner	0.22	-0.85, 1.28	0.70	-0.38, 1.78	0.57	-0.43, 1.57
Physical activities and recreation	Stand up	-0.21	-0.66, 0.24	-0.32	-0.73, 0.08	**-0.45***	**-0.90, -0.01**
	Walk	-0.37	-0.89, 0.15	**-0.53***	**-1.00, -0.06**	**-0.91***	**-1.42, -0.40**
	Exercise	-0.90	-1.40, -0.39	**-1.05***	**-1.51, -0.59**	**-1.18***	**-1.67, -0.68**
	Watch TV	-0.05	-0.69, 0.59	-0.52	-1.10, 0.06	**-0.82***	**-1.45, -0.19**
Social activities	Be alone	**0.67**	**0.13, 1.21**	**0.58***	**0.10, 1.06**	**1.04***	**0.51, 1.57**
	Be with friends	-0.33	-0.90, 0.24	**-0.69***	**-1.21, -0.17**	**-0.98***	**-1.55, -0.42**
	Be with stranger	-0.49	-1.00, 0.03	**-0.58***	**-1.05, -0.12**	**-0.82***	**-1.32, -0.31**
	Blind date	-0.24	-0.80, 0.32	-0.40	-0.90, 0.10	**-0.96***	**-1.50, -0.41**
Hunger and thirst	Hungry	-0.13	-0.67, 0.40	-0.02	-0.50, 0.47	0.05	-0.47, 0.58
	Drink water	0.39	-0.22, 1.01	**-0.63***	**-1.20, -0.06**	0.01	-0.60, 0.62
	Meal	-0.04	-0.55, 0.46	-0.14	-0.59, 0.31	-0.01	-0.50, 0.48
	Fruit	0.09	-0.41, 0.58	-0.12	-0.57, 0.33	0.07	-0.42, 0.56

Food preferences	Sweets/candy	0.03	-0.53, 0.60	0.03	-0.47, 0.52	0.03	-0.51, 0.58
Utilization of resources	Pay money (rank)	3.07	-13.34, 19.48	12.58	-2.18, 27.33	**27.84***	**11.80, 43.88**
	Walk km (rank)	**-20.94***	**-39.99, -1.90**	-3.44	-20.40, 13.52	-3.93	-22.42, 14.57

Table S7. Results of the mixed-effect models for Figure 4 and Figure 5, displaying the coefficient (B), 95% confidence interval (CI), and p-value for each MOSS item with the degree of inflammation (inflammation score) and sleepiness level (Karolinska Sleepiness Scale).

Category	Outcome	Inflammation score			Sleepiness (1-9)		
		b	95% CI	*p*	b	95% CI	*p*
Sleep preparation	At home	**0.27***	**0.03, 0.51**	**0.025**	**0.23***	**0.16, 0.29**	**0.000**
	Close eyes	**0.51***	**0.23, 0.79**	**0.000**	**0.42***	**0.36, 0.48**	**0.000**
	Quiet room	**0.44***	**0.17, 0.70**	**0.001**	**0.23***	**0.15, 0.31**	**0.000**
Resting and sleeping	Rest	**0.34***	**0.06, 0.62**	**0.019**	**0.47***	**0.42, 0.51**	**0.000**
	Sleep own bed	**0.38***	**0.08, 0.68**	**0.012**	**0.48***	**0.43, 0.53**	**0.000**
	Sit	0.24	-0.01, 0.49	0.055	**0.13***	**0.06, 0.21**	**0.001**
Care seeking	Parents	**0.33***	**0.12, 0.54**	**0.003**	**0.10***	**0.02, 0.17**	**0.009**
	Partner	0.24	-0.15, 0.63	0.223	**0.20***	**0.10, 0.31**	**0.000**
Physical activities and recreation	Stand up	-0.09	-0.30, 0.11	0.371	**-0.07***	**-0.13, -0.01**	**0.028**
	Walk	**-0.24***	**-0.48, -0.01**	**0.040**	**-0.19***	**-0.26, -0.13**	**0.000**
	Exercise	**-0.32***	**-0.54, -0.09**	**0.007**	**-0.20***	**-0.27, -0.14**	**0.000**
	Watch TV	-0.25	-0.52, 0.02	0.074	**-0.14***	**-0.23, -0.06**	**0.001**
Social activities	Be alone	**0.34***	**0.07, 0.61**	**0.013**	**0.31***	**0.24, 0.38**	**0.000**
	Be with friends	-0.09	-0.35, 0.16	0.474	**-0.22***	**-0.29, -0.15**	**0.000**
	Be with stranger	-0.19	-0.43, 0.04	0.097	**-0.22***	**-0.28, -0.15**	**0.000**
	Blind date	**-0.27***	**-0.52, -0.02**	**0.037**	**-0.19***	**-0.27, -0.12**	**0.000**
Hunger and thirst	Hungry	-0.11	-0.34, 0.11	0.319	0.06	-0.02, 0.13	0.122
	Drink water	0.05	-0.19, 0.29	0.678	-0.03	-0.11, 0.04	0.365
	Meal	-0.10	-0.31, 0.10	0.317	0.00	-0.06, 0.07	0.949
Food preferences	Fruit	0.01	-0.19, 0.21	0.920	0.02	-0.04, 0.09	0.482
	Sweets/candy	0.01	-0.21, 0.23	0.945	-0.03	-0.10, 0.04	0.384
Utilization of resources	Pay money (rank)	**9.82***	**2.45, 17.20**	**0.009**	**6.93***	**4.81, 9.04**	**0.000**
	Walk km (rank)	-6.02	-13.96, 1.91	0.135	-0.17	-2.73, 2.39	0.895

Note. Asterisks (*) and bold denote statistically significant effects ($p < 0.05$).

Table S8. Results of contrast tests examining the combined effects of vaccination and sleep restriction across all outcome variables**.** Three contrasts were tested for each item: (1) Additivity test ("additive vs combined"): tests whether the combined effect of both challenges equals the sum of the individual effects (null hypothesis = additive effect); (2) Combined vs Vaccine: tests whether the combined condition (vaccine + sleep restriction) differs significantly from the vaccine-only condition; and (3) Combined vs Sleep restriction: tests whether the combined condition differs significantly from the sleep-restriction-only condition.

Category	Outcome	Contrast	b	95% CI	p-value
Sleep preparation	At home	Additivity test	0.11	-0.71, 0.94	0.789
		Combined vs Vaccine	0.62	-0.04, 1.29	0.070
		Combined vs Sleep restriction	0.43	-0.15, 1.02	0.147
	Close eyes	Additivity test	-0.05	-0.93, 0.83	0.913
		Combined vs Vaccine	**1.19***	**0.48, 1.89**	**0.002**
		Combined vs Sleep restriction	0.50	-0.11, 1.11	0.117
	Quiet room	Additivity test	0.26	-0.62, 1.14	0.567
		Combined vs Vaccine	0.32	-0.41, 1.04	0.395
		Combined vs Sleep restriction	0.29	-0.34, 0.92	0.371
Resting and sleeping	Rest	Additivity test	0.74	-0.05, 1.52	0.067
		Combined vs Vaccine	**0.77***	**0.12, 1.41**	**0.021**
		Combined vs Sleep restriction	0.03	-0.52, 0.57	0.926
	Sleep own bed	Additivity test	0.59	-0.26, 1.43	0.174
		Combined vs Vaccine	**1.02***	**0.33, 1.7**	**0.005**
		Combined vs Sleep restriction	0.10	-0.48, 0.69	0.734
	Sit	Additivity test	0.42	-0.48, 1.33	0.358
		Combined vs Vaccine	-0.14	-0.87, 0.58	0.706
		Combined vs Sleep restriction	0.15	-0.50, 0.79	0.657
Care seeking	Parents	Additivity test	0.10	-0.66, 0.86	0.799
		Combined vs Vaccine	0.18	-0.47, 0.83	0.584
		Combined vs Sleep restriction	0.03	-0.50, 0.56	0.923
	Partner	Additivity test	0.36	-1.35, 2.06	0.685
		Combined vs Vaccine	0.35	-0.95, 1.64	0.602
		Combined vs Sleep restriction	-0.14	-1.35, 1.08	0.830
Physical activities and recreation	Stand up	Additivity test	-0.08	-0.76, 0.61	0.825
		Combined vs Vaccine	-0.25	-0.8, 0.3	0.387
		Combined vs Sleep restriction	-0.13	-0.62, 0.36	0.608
	Walk	Additivity test	0.01	-0.8, 0.81	0.989
		Combined vs Vaccine	-0.54	-1.18, 0.11	0.108
		Combined vs Sleep restriction	-0.38	-0.94, 0.19	0.196
	Exercise	Additivity test	-0.77	-1.54, -0.01	0.051
		Combined vs Vaccine	-0.28	-0.88, 0.33	0.380
		Combined vs Sleep restriction	-0.13	-0.68, 0.42	0.651
	Watch TV	Additivity test	0.25	-0.73, 1.23	0.618
		Combined vs Vaccine	-0.77	-1.55, 0.02	0.060
		Combined vs Sleep restriction	-0.30	-1.01, 0.4	0.397
	Be alone	Additivity test	0.21	-0.61, 1.03	0.622

		Combined vs Vaccine	0.37	-0.29, 1.04	0.282
		Combined vs Sleep restriction	0.46	-0.13, 1.05	0.126
		Additivity test	-0.03	-0.9, 0.83	0.938
	Be with friends	Combined vs Vaccine	-0.65	-1.36, 0.05	0.070
		Combined vs Sleep restriction	-0.30	-0.93, 0.33	0.350
Social activities		Additivity test	-0.25	-1.04, 0.53	0.529
	Be with stranger	Combined vs Vaccine	-0.33	-0.96, 0.29	0.305
		Combined vs Sleep restriction	-0.23	-0.8, 0.34	0.420
		Additivity test	0.32	-0.55, 1.18	0.473
	Blind date	Combined vs Vaccine	**-0.72***	**-1.42, -0.01**	**0.049**
		Combined vs Sleep restriction	-0.56	-1.16, 0.05	0.075
		Additivity test	-0.21	-1.03, 0.62	0.621
	Hungry	Combined vs Vaccine	0.19	-0.48, 0.85	0.578
		Combined vs Sleep restriction	0.07	-0.52, 0.66	0.813
Hunger and thirst		Additivity test	-0.25	-1.17, 0.67	0.595
	Drink water	Combined vs Vaccine	-0.38	-1.12, 0.37	0.320
		Combined vs Sleep restriction	0.64	-0.04, 1.33	0.066
		Additivity test	-0.18	-0.94, 0.59	0.654
	Meal	Combined vs Vaccine	0.04	-0.59, 0.66	0.913
		Combined vs Sleep restriction	0.13	-0.42, 0.68	0.637
		Additivity test	-0.11	-0.85, 0.63	0.774
Food preferences	Fruit	Combined vs Vaccine	-0.01	-0.62, 0.6	0.968
		Combined vs Sleep restriction	0.19	-0.35, 0.74	0.481
		Additivity test	0.02	-0.84, 0.89	0.958
	Sweets/candy	Combined vs Vaccine	0.00	-0.7, 0.71	0.991
		Combined vs Sleep restriction	0.01	-0.6, 0.62	0.978
		Additivity test	-12.20	-37.29, 12.89	0.343
	Pay money (rank)	Combined vs Vaccine	**24.78***	**4.34, 45.22**	**0.019**
Utilization of resources		Combined vs Sleep restriction	15.27	-2.48, 33.03	0.096
		Additivity test	-20.52	-49.66, 8.63	0.171
	Walk km (rank)	Combined vs Vaccine	17.05	-6.85, 40.94	0.165
		Combined vs Sleep restriction	-0.46	-20.9, 19.98	0.965
		Additivity test	-0.08	-0.36, 0.20	0.560
	Body temperature	Combined vs Vaccine	-0.02	-0.25, 0.20	0.840
		Combined vs Sleep restriction	**0.33***	**0.13, 0.52**	**0.002**
		Additivity test	**-0.45***	**-0.77, -0.14**	**0.005**
Physiological measures	Inflammation score	Combined vs Vaccine	**0.42***	**0.17, 0.68**	**0.001**
		Combined vs Sleep restriction	**1.39***	**1.17, 1.6**	**0.000**
		Additivity test	0.30	-1.07, 1.67	0.668
	Sleepiness	Combined vs Vaccine	**2.68***	**1.56, 3.8**	**0.000**
		Combined vs Sleep restriction	0.40	-0.58, 1.38	0.416

SI References

1. Lasselin, J., Lekander, M., Benson, S., Schedlowski, M. & Engler, H. Sick for science: experimental endotoxemia as a translational tool to develop and test new therapies for inflammation-associated depression. *Molecular Psychiatry* **26**, 3672–3683 (2020).
2. Palmer, C. A. *et al.* Sleep loss and emotion: A systematic review and meta-analysis of over 50 years of experimental research. *Psychol Bull.* https://doi.org/10.1037/bul0000410 (2023) doi:10.1037/bul0000410.
3. Balter, L. J. T., Sundelin, T., Holding, B. C., Petrovic, P. & Axelsson, J. Intelligence predicts better cognitive performance after normal sleep but larger vulnerability to sleep deprivation. *Journal of Sleep Research* **32**, e13815 (2023).
4. Besedovsky, L. *et al.* Differential effects of an experimental model of prolonged sleep disturbance on inflammation in healthy females and males. *PNAS Nexus* **1**, 1–14 (2022).
5. Banks, S., Van Dongen, H. P. A., Maislin, G. & Dinges, D. F. Neurobehavioral dynamics following chronic sleep restriction: Dose-response effects of one night for recovery. *Sleep* https://doi.org/10.1093/sleep/33.8.1013 (2010) doi:10.1093/sleep/33.8.1013.
6. Gerhardsson, A., Porada, D. K., Lundström, J. N., Axelsson, J. & Schwarz, J. Does insufficient sleep affect how you learn from reward or punishment? Reinforcement learning after 2 nights of sleep restriction. *Journal of Sleep Research* **30**, e13236 (2021).
7. Balter, L. JT. *et al.* Selective effects of acute low-grade inflammation on human visual attention. *NeuroImage* **202**, 116098 (2019).
8. Boyle, C. C. *et al.* Inflammation and dimensions of reward processing following exposure to the influenza vaccine. *Psychoneuroendocrinology* **102**, 16–23 (2019).

9. Brydon, L., Harrison, N. A., Walker, C., Steptoe, A. & Critchley, H. D. Peripheral Inflammation is Associated with Altered Substantia Nigra Activity and Psychomotor Slowing in Humans. *Biological Psychiatry* **63**, 1022–1029 (2008).

10. Balter, L. J. *et al.* Lipopolysaccharide-induced changes in the kynurenine pathway and symptoms of sickness behavior in humans. *Psychoneuroendocrinology* **153**, 106110 (2023).

11. Moieni, M., Irwin, M. R., Jevtic, I., Breen, E. C. & Eisenberger, N. I. Inflammation impairs social cognitive processing: A randomized controlled trial of endotoxin. *Brain, behavior, and immunity* **48**, 132–138 (2015).

12. Bergamaschi, C. *et al.* Systemic IL-15, IFN-γ, and IP-10/CXCL10 signature associated with effective immune response to SARS-CoV-2 in BNT162b2 mRNA vaccine recipients. *Cell Reports* **36**, 109504 (2021).

13. Axelsson, J. *et al.* Sleepiness as motivation: a potential mechanism for how sleep deprivation affects behavior. *Sleep* **43**, zsz291 (2020).

14. Voeten, C. C. Stepwise Elimination and Term Reordering for Mixed-Effects Regression. (2020).
